# PTSD and prospective memory among Afghan students in the Kyrgyz Republic

**DOI:** 10.1192/j.eurpsy.2023.428

**Published:** 2023-07-19

**Authors:** E. Molchanova

**Affiliations:** Psychology, American University of Central Asia, Bishkek, Kyrgyzstan

## Abstract

**Introduction:**

Prospective memory is a memory for actions to be performed in the future, such as composing an abstract for Congress. Various studies (see, for instance, Khan, A, 2020) showed that deficits in prospective memory are part of depressive cognitive deficits. This study is devoted to the prospective memory characteristics of migrants - students from Afghanistan who study at American University in Central Asia

**Objectives:**

The study’s objective was to test the hypothesis of the existing connections between PTSD and prospective memory.

**Methods:**

One hundred and fifty students submitted informed consent for participation in the study, which the local IRB approved. Twenty-five had been diagnosed with PTSD; others also experienced traumatic stress but did not present the complete clinical picture of PTSD.

The research was quantitative; a variety of self-questioners were used, including the one Dr developed by Azzizudin Khan in 2021 (Khan and others, 2021) to measure traumatic stress level, depression, anxiety, and subjective perception of the prospective memory.

ANOVA and family of regression statistics helped to establish connections between several variables.

One hundred and fifty students submitted informed consent for participation in the study, which the local IRB approved. Twenty-five had been diagnosed with PTSD; others also experienced traumatic stress but did not present the complete clinical picture of PTSD. The research was quantitative; a variety of self-questioners were used, including the one Dr developed by Azzizudin Khan in 2021 (Khan and others, 2021) to measure traumatic stress level, depression, anxiety, and subjective perception of the prospective memory. ANOVA and family of regression statistics helped to establish connections between several variables.

**Results:**

The study’s preliminary results, which are still in progress, showed that the perceived level of traumatization predicts the perceived failure in prospective memory. However, there are also a lot of statistical outcomes which need to be analyzed. Among those are, for instance, the connection between depression and prospective memory and the connection between prospective memory and anxiety.

**Conclusions:**

Prospective memory deficiency is a part of a traumatic cognitive deficit. More research is needed to investigate cognitive distortion in PTSD.

**Disclosure of Interest:**

None Declared

